# Hydralazine-induced antineutrophil cytoplasmic antibody vasculitis with significant dermatologic manifestations

**DOI:** 10.1016/j.jdcr.2025.12.039

**Published:** 2026-01-27

**Authors:** Aanal Patel, Mahroo Tajalli, Amit Singal, Lisa Zhou, Cindy Wassef

**Affiliations:** a Department of Internal Medicine, Temple University Hospital, Philadelphia, Pennsylvania; b Department of Dermatology, Rutgers Robert Wood Johnson Medical School, New Brunswick, New Jersey; c New Jersey Medical School, Newark, New Jersey

**Keywords:** ANCA-associated vasculitis, drug reaction, hydralazine

## Case description

Our department was consulted for a new purpuric rash in a 71-year-old woman with hypertension and heart failure with reduced ejection fraction. She had been hospitalized for angioedema of unclear etiology and subsequently developed low-grade fever, leukocytosis, and thrombocytopenia, followed by a rapidly progressive rash.

**Fig 1:**
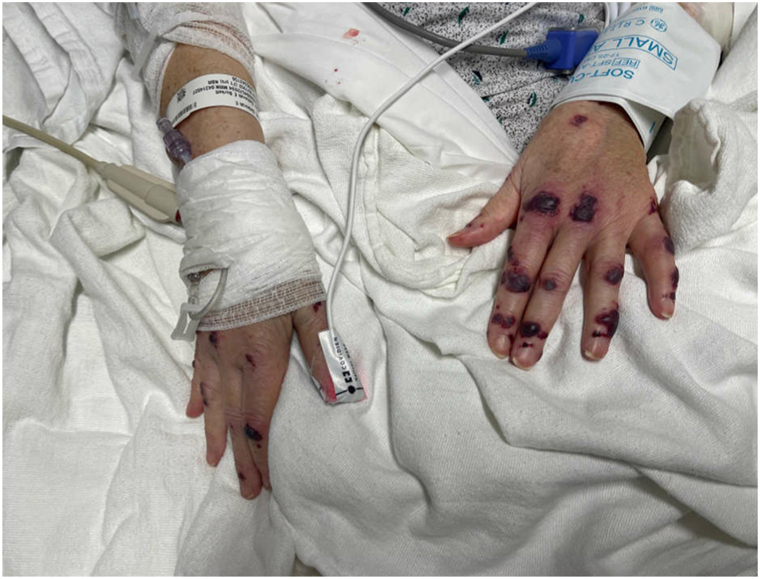
Hemorrhagic bullae over bilateral hands.

**Fig 2:**
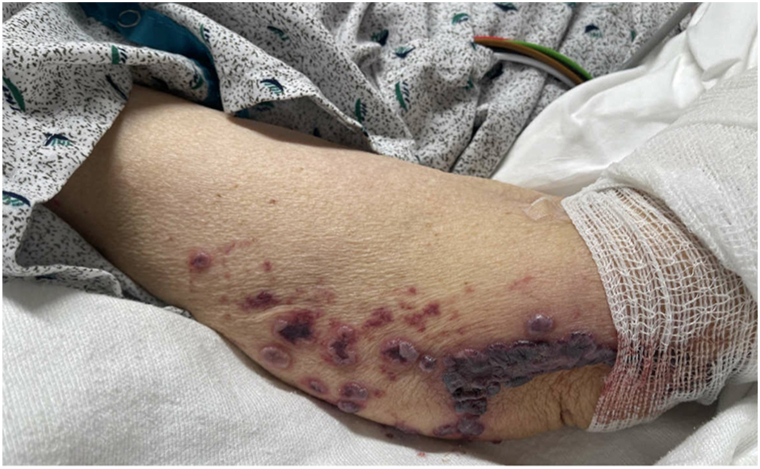
Hemorrhagic bullae over left elbow.

**Fig 3:**
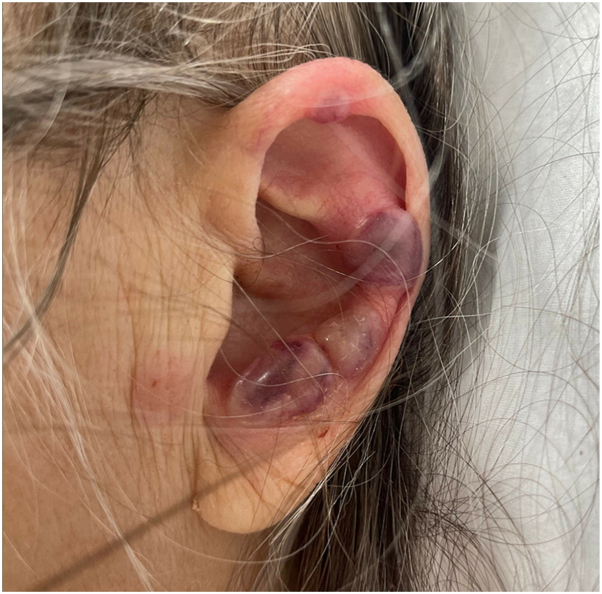
Hemorrhagic bullae over left ear.

**Fig 4:**
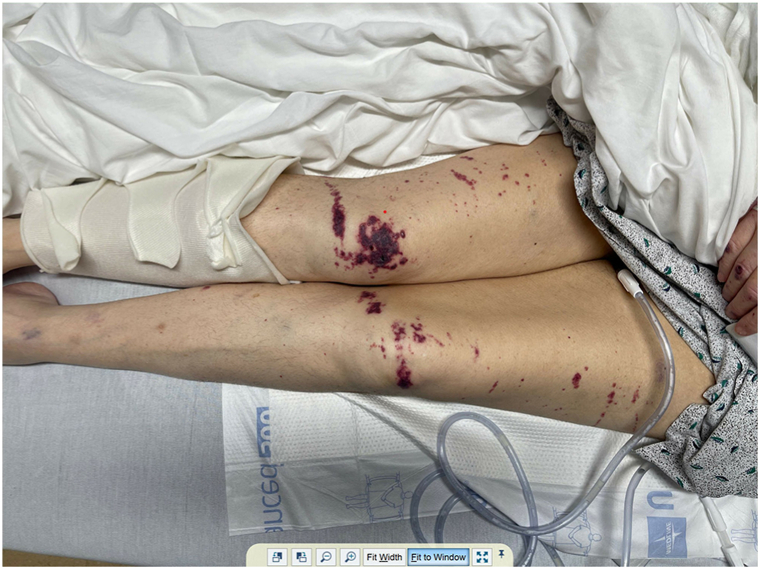
Purpuric papules, plaques and hemorrhagic bullae over lower extremities.

Examination revealed widespread purpuric plaques and hemorrhagic bullae affecting the eyelids, nasal tip, ears, and extremities (Fig 1, Fig 2, Fig 3 to 4). Oral examination showed erosions and hemorrhagic crusting of the palate; genital mucosa was spared.

Laboratory studies showed elevated inflammatory markers, antinuclear antibodies 1:1280 (homogeneous), positive p-antineutrophil cytoplasmic antibody (ANCA) and c-ANCA, elevated myeloperoxidase (MPO)-ANCA (159 U) and proteinase 3-ANCA (1.1 U), and positive anti-histone antibodies. Rheumatoid factor, anticardiolipin IgM, lupus anticoagulant, and type III cryoglobulins were positive, with low C3 and undetectable C4. Anti-double-stranded DNA, anti-Smith, hepatitis B/C serologies, and protein electrophoresis were negative. Chest computed tomography revealed bilateral lower lobe atelectasis with moderate pleural effusion. A review of her home medications revealed chronic use of hydralazine 100 mg twice daily for 5-years for the management of hypertension and heart failure with reduced ejection fraction.

**Fig 5:**
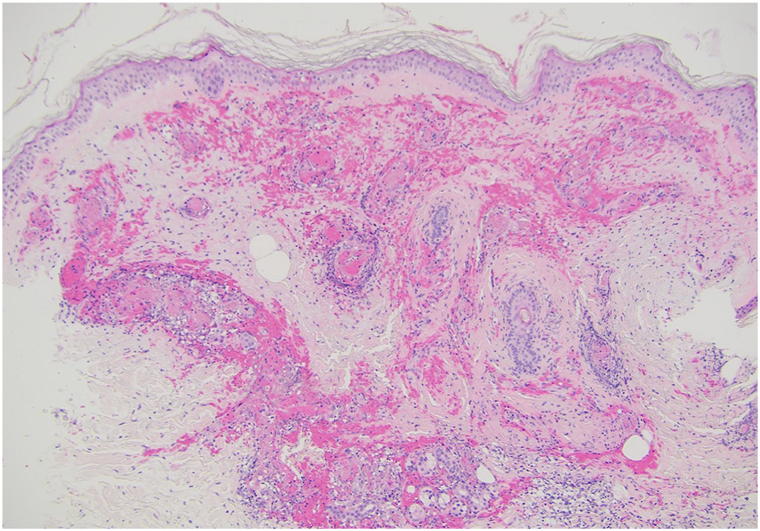
Leukocytoclastic vasculitis, microthrombi in some vessel lumens, neutrophilic infiltrate in the dermis and upper part of the subcutis, and numerous extravasated erythrocytes.

Skin biopsy demonstrated leukocytoclastic vasculitis with neutrophilic infiltration, vessel wall thrombosis, and extravasated erythrocytes; direct immunofluorescence supported immune-mediated vasculitis (Fig 5).

Hydralazine was promptly discontinued. Given poor response to plasmapheresis, treatment was escalated to high-dose intravenous corticosteroids and rituximab, followed by a gradual steroid taper. Shortly after tapering, the patient developed melena secondary to gastrointestinal vasculitis. Despite IR embolization and intensified corticosteroid therapy, she experienced catastrophic gastrointestinal hemorrhage with disseminated intravascular coagulation and succumbed to fulminant systemic vasculitis.

**Question: A patient on chronic hydralazine presents with purpura, oral ulcerations, and positive antinuclear antibodies and antihistone antibodies. Which condition is most important to distinguish from hydralazine-induced ANCA vasculitis?**

**A.** Systemic lupus erythematosus
**B.** Stevens-Johnson syndrome
**C.** Cryoglobulinemic vasculitis
**D.** Bullous pemphigoid
**E.** Drug reaction with eosinophilia and systemic symptoms

**Answer: A. Systemic lupus erythematosus.**

## Discussion

Hydralazine, a selective arteriolar vasodilator used to treat hypertension, hypertensive emergencies in pregnancy, and heart failure with reduced ejection fraction, is generally safe and well tolerated.^1^ Rarely, it can induce hydralazine-induced ANCA-associated vasculitis—a potentially life-threatening autoimmune reaction.^1^ This condition involves inflammation and necrosis of small blood vessels, presenting with purpura, arthralgias, myalgias, fever, and multiorgan involvement.^2^

Hydralazine may trigger autoimmunity through several mechanisms. It may deplete neutrophilic histones at MPO and proteinase 3 loci, causing gene demethylation and antigen expression, or inhibit DNA methyltransferase, unmasking previously hidden antigens. It may also act as a hapten, binding MPO, and eliciting an immune response.^1^^,^^3^

Hydralazine-induced ANCA vasculitis presents variably, most often affecting the kidneys, lungs, and skin. Cutaneous manifestations such as palpable purpura and urticaria result from vascular inflammation and leakage. Severe cases may lead to mucosal ulcerations, limb ischemia, or fatal multiorgan failure.^2^, ^3^, ^4^ Our case demonstrated rare complications, including angioedema, pleural effusion, oral ulcerations, and gastrointestinal bleeding—features infrequently reported.^1^^,^^3^

Hydralazine-induced vasculitis can mimic systemic lupus erythematosus due to overlapping clinical and serologic features. Unlike systemic lupus erythematosus, it is typically characterized by positive ANCA and antihistone antibodies with negative anti-double-stranded DNA,^3^ making serologic testing essential for accurate diagnosis.

There is no standardized treatment; and management depends on severity. Prompt discontinuation of hydralazine is critical, with systemic corticosteroids or immunosuppressive therapy indicated for organ-threatening disease.^5^ This case underscores the importance of early recognition, vigilance for atypical presentations, and the development of standardized treatment strategies to improve outcomes.

## Conflicts of interest

None disclosed.
